# Effects of Astaxanthin on Miniature Pig Sperm Cryopreservation

**DOI:** 10.1155/2018/6784591

**Published:** 2018-04-19

**Authors:** Eunjoo Lee, Daeyoung Kim

**Affiliations:** Department of Life Sciences, College of Bio-Nano Technology, Gachon University, Seongnam 13120, Republic of Korea

## Abstract

The purpose of this study is to evaluate the effects of astaxanthin added to freezing buffer on semen parameters, total sperm oxidation stress after postthawing of boar sperm, and lipid peroxidation (LPO) which is caused by reactive oxygen species (ROS) in sperm membrane. Varying concentrations of astaxanthin (0, 10, 50, 100, and 500 *μ*M) were used in the freezing buffer during cryopreservation to protect the DNA of thawed miniature pig sperm. Semen parameter was measured using computer-assisted sperm analysis (CASA) for sperm motility, and then ROS rate and oxidative stress of boar sperm were determined using fluorescence-activated cell sorting (FACS). Sperm motility was higher (*p* < 0.05) in the astaxanthin group than in the control group. Sperm motility and the number of progressive motile sperm were higher (*p* < 0.05) in the astaxanthin 500 *μ*M group than in the control group. In ROS evaluation, the astaxanthin group had lower intracellular O_2_ and H_2_O_2_ in viable sperm. Yo-Pro-I/HE and PI/H2DCFDA staining as revealed using flow cytometry was lower in astaxanthin groups than in the other groups. As a result, we found that astaxanthin could protect the sperm plasma membrane from free radicals and LPO during boar sperm postthawing.

## 1. Introduction

Thanks to the development of biotechnology and the area of biomedicine, stem cell culture techniques and technologies for the production of animals for organ transplantation and the mass production of therapeutic materials using transgenic animals have developed [1]. Meanwhile, miniature pigs produced using new technologies are anatomically and physiologically similar to humans and thus are used as experimental animals for studies on biomedicine and are receiving attention as animals for interspecific organ transplantation.

In the case of miniature pigs mostly produced through natural mating, difficulties are being experienced in securing sires and producing litters. Therefore, artificial insemination (AI) is necessary to improve the productivity of miniature pigs and to use good sires. Methods of using semen for AI include using liquid sperm and preserving sperm as frozen sperm. Although AI using liquid sperm is universally used, due to the limitation of this method that sperm can be preserved only for a short period of time, frozen storage methods that can supplement the limited longevity* in vitro* of collected semen are being reported [2].

Although frozen storage is an efficient technology, it causes damage to cell membranes, abnormal enzyme activity, and damage to DNA due to the cold shock resulting from drastic temperature changes [3–9]. In addition, due to the increase in reactive oxygen species (ROS) occurring in freezing procedures, sperm motility, survivability, fertilizing capacity, and functions decline after freeze-thawing [10].

Although AI using frozen sperm is commonly used in the case of cattle, it cannot be easily used in clinics in the case of pigs because artificial fertilization rates are low since the oxidative substances formed after freeze-thawing cause stress to sperm [11–14]. Compared to other livestock species, pig semen responds to harmful active oxygen more sensitively because pig sperm contain higher ratios of unsaturated fatty acid in their cell membranes but have relatively fewer antioxidants. Frozen storage has been reported to promote sperm cell membrane lipid oxidation and drastically reduce sperm motility and survivability [15].

Antioxidants remove oxidative substances in sperm and fight against ROS to suppress sperm cell membrane lipid oxidation, thereby enabling sperm to maintain their shapes and functions [16, 17]. Recently, studies indicating that diverse antioxidants can mitigate lipid oxidation occurring due to active oxygen have been actively conducted [18]. *α*-Tocopherol, albumin, ascorbic acid, taurine, and hypotaurine are known as antioxidants that mitigate ROS that causes oxidation in sperm cell membranes. Among them, *α*-tocopherol (vitamin E) mainly exists in the cell membrane and suppresses lipid oxidation as it has antioxidant functions. Although *α*-tocopherol's functions in cells are well known, their functions in sperm are not known very well. Taurine and hypotaurine play the role of protecting sperm from lipid oxidation [19–22]. Meanwhile, astaxanthin (AXT, C_4_0H_52_O_4_, MW 596.84), which is a carotenoid component known as a powerful antioxidative substance, is a natural carotenoid antioxidant that can be extracted from crustaceans or microalgae and is a brilliant red lipid-soluble substance. AXT has a strong antioxidative activity with a better ability to remove O_2_ compared to vitamin E [23, 24], and it plays the role of preventing lipid oxidation from occurring in cell membranes [25–27].

Although many studies of AXT have been reported, results that demonstrate their antioxidative effects are still insufficient. Therefore, in the present study, to establish stable frozen storage methods for mass propagation of miniature pigs, attempts were made to examine the effects of AXT as antioxidants added to frozen storage solutions during manufacturing on sperm survivability, sperm cell membranes' integrity, ROS occurrence, and lipid oxidation in sperm cells after thawing.

## 2. Materials and Methods

### 2.1. Chemicals

All chemicals were purchased from Sigma-Aldrich if not mentioned otherwise.

### 2.2. Semen Collection

The pig semen used in the present study was collected from PWG miniature pigs being raised at Kangwon National University through a penis hand pressing method using a dummy and was transported to the laboratory in Incheon while being maintained at 17°C. Using computer-assisted sperm analysis (CASA, Hamilton Thorne, Inc., HTM-HELOS, Beverly, MA, USA), only those semen samples with 80% or higher sperm motility and 70% or higher anterograde movements were used.

### 2.3. Astaxanthin Preparation

AXT (3,3′-dihydroxy-*β*,*β*-carotene-4,4′-dione), known as a high-value carotenoid pigment extracted from green algae especially from* Haematococcus* species, has widely been studied in several fields including food supplements, cosmetics, and pharmaceutical industries. The photosynthetic unicellular green algae* H. lacustris* (UTEX 16) were purchased from the Culture Collection of Algae at the University of Texas at Austin. To obtain microalgal biomass, a single colony of* H. lacustris* grown on an agar plate was cultured in modified Bold's basal medium (MBBM) as described in a previous study [23]. Compact fluorescent lamps (Model DULUX L®, OSRAM Korea, Ansan, Korea) were used for photobioreactors as the external illumination. The cells were then inoculated at a density of 2 × 10^4^ cells mL^−1^ into 500 mL of bubble column photobioreactor containing 400 mL of culture broth, providing 0.2 v/v/m (volume per volume per minute) aeration containing 5% CO_2_ gas and 95% air under constant continuous light irradiance as described above. Samples were collected and analyzed daily to observe the process of the cultures and AXT accumulation in* H. lacustris* cells. The concentration of AXT following acetone extraction of AXT from the cells was estimated by a calibration curve using synthetic AXT (A9335, Sigma Chemical Co., St. Louis, MO, USA) as a standard following the calibration: AXT concentration (mg L^−1^) = 0.0045 × OD475. For* in vitro* assay, AXT was extracted from the cells using a simple process. Briefly, cells (1.0 g) were placed in a mortar and frozen with liquid nitrogen (50 mL). Using a mortar bar, cells were crushed and destroyed by hand grinding. Freezing-thawing-grinding processes were repeated five times and cells were suspended in ethanol (99%) to extract AXT from the crushed cells. The concentration of AXT was measured following the simple equation described above [28, 29].

### 2.4. Diluent and Frozen Storage Solution

For the pig semen, modified Modena B (mMB: 6 g glucose, 0.45 g EDTA, 1.38 g sodium citrate, 0.2 g sodium bicarbonate, 1 g Tris base, 0.5 g citric acid, 0.01 g cysteine, 0.8 g BSA, and 0.06 g kanamycin sulfate, pH 7) was used as a basic diluent, lactose-egg yolk (LEY extender: 80% v/v lactose solution [310 mM] and 20% v/v egg yolk, 100 *μ*g mL^−1^ kanamycin sulfate) was used as a primary frozen storage solution, and LEY-glycerol-Orvus-ES-Paste (LEYGO: 89.5% v/v LEY; 9% v/v glycerol; 1.5% v/v Orvus ES Paste (OEP, Nova Chemical Sales, Inc., Scituate, MA); 100 mM trehalose; and 0, 10, 50, 100, and 500 *μ*M of AXT) was used as a secondary frozen storage solution.

### 2.5. Preparation of Pig Semen

The pig semen was diluted with mMB solution at a ratio of 1 : 3 and subjected to centrifugation (400 ×g, 10 min, 24°C). The semen was washed using diluted mMB solution 3–5 times depending on semen conditions. After washing, the semen was diluted with LEY so that the number of sperm became 1 × 10^9^/mL and was quantified. The sperm-LEY solution was cooled to become 4°C by gradually reducing its temperature at a rate of 1°C per five minutes in ice water. Thereafter, the solution was mixed with LEYGO added with AXT at concentrations of 0, 10, 50, 100, and 500 *μ*M, respectively, at a ratio of 1 : 1. The sperm-LEYGO mixture solutions were put into 0.5 mL straws, prefrozen for five minutes with the chill of liquid nitrogen at approximately 5 cm above a Styrofoam box containing liquid nitrogen, and stored in liquid nitrogen.

### 2.6. Thawing of Frozen Pig Semen and Sperm Motility Test

Sperm motility was measured through computer-assisted sperm analysis (CASA). The straws kept frozen were thawed by immersion in a constant-temperature water tank preheated to 50°C for 10 seconds, put into 15 mL tubes, diluted with 10 mL of mMB, and left in a constant-temperature water tank preheated to 37°C for 5–10 min. When the temperature of the diluted sperm became 37°C, the solution was subjected to centrifugation (13,000 rpm, 5 min) and diluted so that the number of sperm became 5 × 10^7^ mL^−1^ by adding mMB. Two microliters of the sperm was put into a Leja slide chamber (Leja Products BV, Nieuw-Vennep, Netherlands) preheated to 37°C and observed using a microscope. Using CASA analysis, the motility, progressive motility, VAP (path velocity), VSL (straight-line velocity), and VCL (curvilinear velocity) were observed.

### 2.7. Flow Cytometry for Oxidative Substances and Their Distributions in Pig Sperm

The number of extinct sperm and the quantities of oxidative substances in sperm by the concentration of AXT (provided by Prof. Jaekweon Park, Department of Life Science, Gachon University) added when the sperm were being frozen were measured with FACS (500 series, Beckman Coulter, Inc., FL, USA) using two fluorescent staining methods. Yo-Pro-1 iodide (Yo-Pro-1; Molecular Probes Inc.) 0.05 *μ*M and hydroethidine (HE; Molecular Probes Inc., Eugene, OR, USA) 4 *μ*M were added to each of the solutions at individual concentrations of AXT solutions and the solutions were made to react for 40 min at 25°C and used to measure the quantities of O_2_^−^ in the sperm. Thereafter, propidium iodide (PI; Sigma-Aldrich) 0.75 *μ*M and 2′,7′-dichlorodihydrofluorescein diacetate (H_2_DCFDA; Molecular Probes Inc.) 200 *μ*M were added to each of the solutions and the solutions were made to react for 10 min at 37°C and used to measure the quantity of H_2_O_2_ in the sperm.

### 2.8. Statistical Processing

All the data used in the present experiments were statistically processed by conducting variance analyses using the R statistical program (R Development Core Team). The significant difference of the data values obtained through the statistical processing is *p* < 0.05.

## 3. Results

### 3.1. Pig Sperm Motility Test (CASA)

The effects of the addition of AXT when frozen storage solutions of pig semen were made on the motility the sperm are as shown in Table 1. When the frozen storage solutions were made with the addition of AXT, the motility of the sperm was shown to be generally higher than that of the control group. The ratios of sperm that showed movements (motility) were shown to be higher in the AXT 50 *μ*M, AXT 100 *μ*M, and AXT 500 *μ*M groups than in the control group. Progressive motility was shown to be higher in the AXT 50 *μ*M, AXT 100 *μ*M, and AXT 500 *μ*M groups than in the control group. In addition, VAP (average-path velocity), VSL (straight-line velocity), and VCL (curvilinear velocity) were also shown to be higher in the AXT 500 *μ*M group than in the control group.

### 3.2. Analysis of Oxidative Substances in Pig Sperm (FACS)

**Figure 1:**
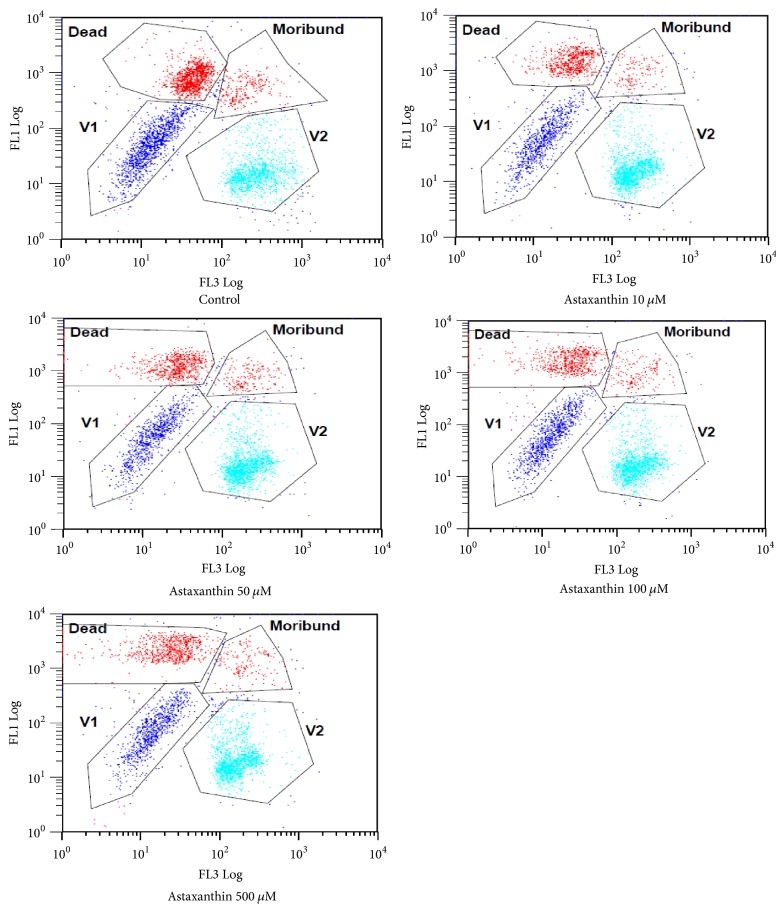
Flow cytometric analysis of sperm labeled with Yo-Pro-1/HE fluorescence in control and AXT treated groups. V1 region represents viable sperm with a low intracellular O_2_ radical, and V2 region represents viable sperm with a high intracellular O_2_ radical, and the upper quadrant Dead and Moribund regions represent dead sperm, in the process of programmed cell death of sperm. Yo-Pro-1/HE fluorescence: measure O_2_ radical of sperm. Yo-Pro-1: FL1 Log; HE: FL3 Log.

**Figure 2:**
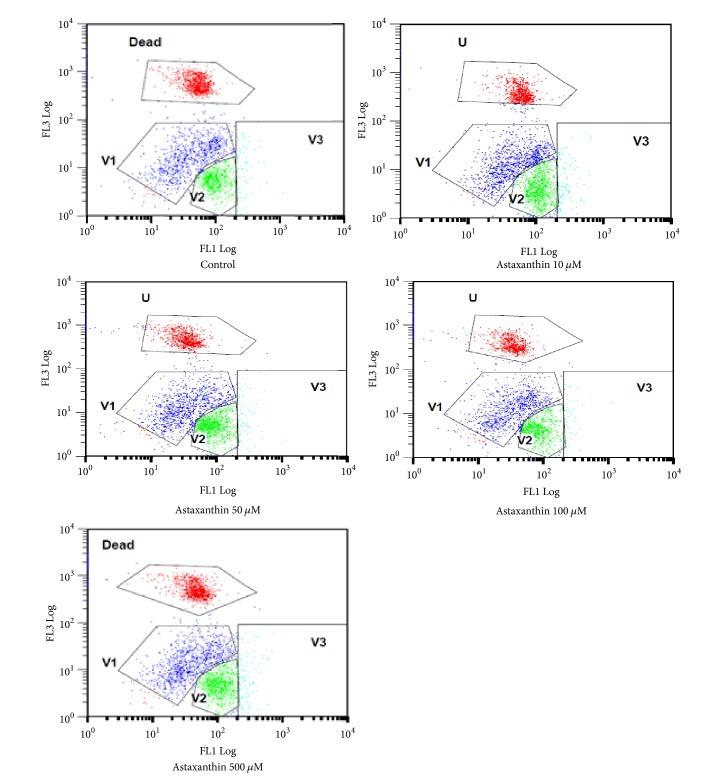
Flow cytometric analysis of sperm labeled with PI/H_2_DCFDA fluorescence in control and AXT treated groups. V1 and V2 regions represent viable sperm with a low intracellular H_2_O_2_ radical, and V3 region represents viable sperm with a high intracellular H_2_O_2_ radical, and the upper quadrant Dead region represents dead sperm. PI/H_2_DCFDA fluorescence: measure H_2_O_2_ of sperm. PI: FL1 Log; H_2_DCFDA: FL3 Log.

The following are the results of FACS analysis of pig sperm added with AXT after being stained using Yo-Pro-1 and HE (Figure 1). The O_2_^−^ values in the cells can be seen from the ratios of cells located in the V2 area. Therefore, the experimental groups added with AXT showed significantly higher values than the control group. In addition, H_2_O_2_ in pig sperm cells can be identified by staining pig sperm added with AXT using PI and H_2_DCFDA and analyzing the stained pig sperm using FACS (Figure 2). When V3 areas where cells containing many H_2_O_2_ are located were compared with each other, the ratios of V3 area cells in the control group and the experimental groups were shown to be similar except for the AXT 100 *μ*M group that showed a significantly lower value.

### 3.3. Analysis of ROS in Pig Sperm

**Figure 3:**
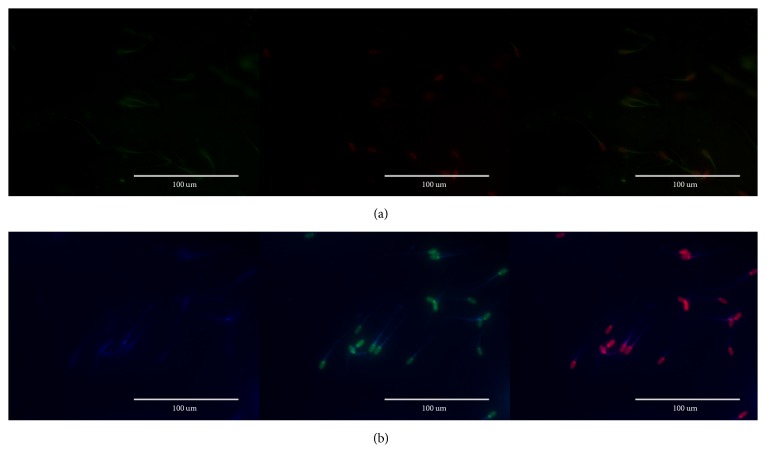
Microscopic view of sperm stained with Yo-Pro-1/HE (a) or PI/H_2_DCFDA (b) fluorescence (EVOS^®^ FL, Thermo Fisher Scientific). The images indicate apoptotic cells (green), whereas dead nonapoptotic cells are red in color. Four populations of sperm were obtained: light green or clear (live cells), dark green (apoptotic cells), red (dead/necrotic cells), and red and green (dead cells).

ROS levels were examined through two different fluorescent probes: hydroethidine (HE) and 2′,7′-dichlorodihydrofluorescein diacetate (H_2_DCFDA), used to evaluate the intracellular content of superoxide anions (O_2_^−∙^) and peroxides (H_2_O_2_), respectively. After incubation, 2 *μ*L of the stained sperm was placed on warmed slide glass and examined under a fluorescent microscope (EVOS FL, Thermo Fisher Scientific). We divided sperm staining into four different groups: light green or clear (live cells), dark green (apoptotic cells), red (dead/necrotic cells), and red and green (dead cells) cells (Figure 3).

## 4. Discussion

Semen cryopreservation is an important axis of animal reproductive biotechnology, but its potential must still be realized as much of the mammalian sperm lose fertility in the freezing and thawing process. Cryopreservation of sperm leads to overproduction of reactive oxygen species (ROS), mainly by heat shock, exposure to atmospheric oxygen, and formal plasma elimination. Subsequent oxidative damage can cause significant changes in sperm motility behavior, membrane integrity, lipid peroxidation (LPO), DNA damage, and apoptosis, ultimately reducing fertility [30].

Although frozen storage methods are highly efficient, they are accompanied by drastic temperature changes. Pig sperm are easily affected by cold shocks and are not sufficiently resistant to low temperatures. Therefore, they are highly likely to become extinct due to differences in intracellular osmotic pressure or damage to DNA in cell nuclei [3, 13]. Ram, rabbit, and pig sperm are severely damaged during the freeze-thawing process and such damage reduces sperm motility [31–33]. Sperm cell membranes have unsaturated fatty acid that can be changed into lipid peroxides. The sperm damaged by the active oxygen generated in the process of sperm freezing are subjected to oxidative stress which causes damage to sperm structures leading to reduced sperm motility [34]. Antioxidative substances prevent lipid oxidation from occurring in sperm cell membranes so that sperm can maintain their metabolic activity and functions. This enhances the survival rates of sperm and maintains their motility after freeze-thawing, thereby enhancing the utility of frozen sperm [35].

Recently, studies have been conducted on the addition of antioxidants during the manufacturing of frozen sperm to improve sperm functions after freeze-thawing. *α*-Tocopherol, taurine, hypotaurine, and trehalose are known as antioxidants that mitigate ROS that causes oxidation in bovine, goat, rabbit, ram, and rat sperm cell membranes [36, 37]. Taurine plays the role of protecting sperm from active oxygen when sperm are exposed to oxygen or during the process of freeze-thawing. Hypotaurine plays the role of protecting sperm from lipid oxidation. Trehalose increases sperm resistance to damage during freeze-thawing through interactions with phospholipid in sperm cell membranes. Among them, *α*-tocopherol (vitamin E) mainly exists in cell membranes and suppresses lipid oxidation because it has antioxidant functions. Although *α*-tocopherol's functions in cells are well known, their functions in mammalian sperm are not well known [20–22].

The experimental data indicate that when treated with AXT, higher ratios of sperm survived and showed movements in the freeze-thawing process. When the motility of the sperm was analyzed using CASA, it could be seen that the motility was highest when the sperm were treated with AXT at a concentration of 500 *μ*M.

In the current study, the AXT which are next-generation antioxidative components have been attracting great attention in the entire bioindustry such as food, medicines, and cosmetics industries. The major function of AXT is antioxidative activity. Its ability to remove O_2_ is excellent to the extent that it is comparable to that of *α*-tocopherol and it plays the role of preventing lipid oxidation from occurring in cell membranes [25]. Astaxanthin suppresses damage to intracellular DNA, proteins, and lipids by active oxygen as well as tissue aging and carcinogenesis and plays the role of suppressing the formation of free radicals [27].

In summary, the results suggest that all experimental groups added with AXT showed significantly higher motility and progressive motility compared to the control group (*p* < 0.05) (Table 1). According to the results of analyses of oxidative substances in pig sperm conducted using FACS, the experimental groups added with AXT had generally lower values than the control group but did not have any significantly better ability to remove O_2_ and H_2_O_2_ (Table 2). Given that the formation of H_2_O_2_ was suppressed in the patterns of formation of active oxygen in the experimental groups added with AXT, the effects of H_2_O_2_ seem to account for more of the harmfulness of freezing of pig sperm than do the effects of O_2_.

Based on the results of the present experiments, the addition of astaxanthin as antioxidants to frozen storage solutions in the manufacturing of frozen miniature pig semen protected sperm from damage in the freeze-thawing process, maintained sperm motility, and prevented lipid oxidation to improve sperm metabolic activity and functions.

## 5. Conclusion

It could be seen that astaxanthin added to frozen storage solutions when frozen miniature pig semen was manufactured improved sperm motility and antioxidative effects. The experimental groups added with AXT showed significantly higher motility compared to the control group. In particular, in the experimental group added with AXT at a concentration of 500 *μ*M, 66 ± 1.7% of sperm showed movements and 45.7 ± 2.5% of sperm showed normal movements. In addition, the experimental group added with AXT at a concentration of 100 *μ*M showed the highest efficiency in removing free radicals. The results of the present study are thought to be importantly used in the manufacturing of frozen miniature pig sperm for increasing reproductive cells and livestock propagation. Future studies utilizing diverse additives and antioxidants for improving sperm functions after freeze-thawing are considered necessary.

## Figures and Tables

**Table 1:** Effects of astaxanthin (AXT) on standard sperm parameters (mean ± SD).

Sperm treatment	Motility (%)	Progressive motility (%)	VAP (*μ*ms^−1^)	VSL (*μ*ms^−1^)	VCL (*μ*ms^−1^)
Fresh	93.4 ± 0.6^c^	71.0 ± 9.2^c^	100.3 ± 15.6	70.1 ± 15.7	182.6 ± 30.8
Control	49.8 ± 4.0^a^	33.4 ± 2.5^ab^	99.3 ± 9.6	63.4 ± 7.7	180.9 ± 12.5
AXT 10 *μ*M	42.0 ± 11.8^a^	27.0 ± 7.2^a^	92.7 ± 4.5	58.7 ± 1.7	178.2 ± 10.3
AXT 50 *μ*M	55.7 ± 2.5^ab^	35.7 ± 2.1^ab^	99.7 ± 9.6	60.4 ± 5.6	195.5 ± 15.9
AXT 100 *μ*M	52.7 ± 3.0^ab^	31.0 ± 5.3^ab^	98.5 ± 5.0	56.6 ± 7.6	192.9 ± 9.2
AXT 500 *μ*M	66.0 ± 1.7^b^	45.7 ± 2.5^b^	110.4 ± 11.5	70.8 ± 5.9	203.4 ± 26.0

VAP: average-path velocity; VSL: straight-line velocity; VCL: curvilinear velocity. The results are expressed as means ± SEM of a total number of analyzed boar sperm of 14660. Motility means sperm's ability to move spontaneously and actively, consuming energy in the process; progressive motility means sperm's ability to swim fast in a straight line; VAP means the mean velocity of the sperm head along its average trajectory; VSL means the mean path velocity of the sperm head along a straight line from its first to its last position; VCL means the mean path velocity of the sperm head along its trajectory. a, b, and c indicate difference *P* < 0.05.

**Table 2:** ROS evaluation of ethidium and DCF fluorescence in frozen-thawed boar sperm of control and astaxanthin (AXT).

Sperm treatment	Viable sperm with high O_2_^−^ (%)	MFI of ethidium/total sperm	MFI of ethidium/viable sperm	Viable sperm with high H_2_O_2_^−^ (%)	MFI of DCF/total sperm	MFI of DCF/viable sperm
Control	31.5 ± 1.8^a^	116.8 ± 20.4	135.0 ± 24.0	4.0 ± 0.8	72.2 ± 0.9^ab^	87.7 ± 2.1^ab^
AXT 10 *μ*M	44.4 ± 1.⁡0^c^	106.9 ± 22.8	114.0 ± 23.0	4.8 ± 0.9	83.0 ± 8.2^b^	95.7 ± 7.2^b^
AXT 50 *μ*M	43.7 ± 1.6^bc^	118.0 ± 6.6	126.6 ± 13.8	4.0 ± 0.8	73.8 ± 6.8^ab^	87.8 ± 8.0^ab^
AXT 100 *μ*M	41.1 ± 0.9^bc^	116.7 ± 10.5	121.2 ± 9.9	2.7 ± 0.9	65.6 ± 4.3^a^	76.0 ± 4.5^a^
AXT 500 *μ*M	40.8 ± 0.1^b^	124.3 ± 16.1	130.7 ± 16.4	4.3 ± 0.3	78.0 ± 2.3^ab^	95.1 ± 2.4^b^
*p* value	*p* < 0.001	*p* > 0.05	*p* > 0.05	*p* > 0.05	*p* < 0.05	*p* < 0.01

a, b, and c indicate difference *P* < 0.001, *P* < 0.05, and *P* < 0.01, respectively.
